# The Comprehensive Post-Acute Stroke Services (COMPASS) study: design and methods for a cluster-randomized pragmatic trial

**DOI:** 10.1186/s12883-017-0907-1

**Published:** 2017-07-17

**Authors:** Pamela W. Duncan, Cheryl D. Bushnell, Wayne D. Rosamond, Sara B. Jones Berkeley, Sabina B. Gesell, Ralph B. D’Agostino, Walter T. Ambrosius, Blair Barton-Percival, Janet Prvu Bettger, Sylvia W. Coleman, Doyle M. Cummings, Janet K. Freburger, Jacqueline Halladay, Anna M. Johnson, Anna M. Kucharska-Newton, Gladys Lundy-Lamm, Barbara J. Lutz, Laurie H. Mettam, Amy M. Pastva, Mysha E. Sissine, Betsy Vetter

**Affiliations:** 1 Department of Neurology, Wake Forest School of Medicine, Medical Center Blvd, Winston-Salem, NC 27157 USA; 2 Department of Epidemiology, University of North Carolina at Chapel Hill, Gillings School of Global Public Health, 135 Dauer Drive, Chapel Hill, 27599 USA; 3 Department of Social Sciences & Health Policy, Division of Public Health Sciences, Wake Forest School of Medicine, Winston-Salem, NC 27157 USA; 4 Department of Biostatistical Sciences, Division of Public Health Sciences, Wake Forest School of Medicine, Winston-Salem, NC 27157 USA; 5 Piedmont Triad Regional Council Area Agency on Aging, 1398 Carrollton Crossing Drive, Kernersville, NC 27284 USA; 6 Duke University School of Medicine, 40 Medicine Circle DUMC 2919, Durham, NC 27710 USA; 7 East Carolina University, Brody School of Medicine, Family Medicine Center, MS #654, 101 Heart Drive, Greenville, NC 27834 USA; 8 Department of Physical Therapy, University of Pittsburgh, Bridgeside Point 1, 100 Technology Drive, Suite 210, Pittsburgh, PA 15219-3130 USA; 9 Department of Family Medicine, University of North Carolina at Chapel Hill, 725 Martin Luther King Jr. Blvd., CB #7590, Chapel Hill, NC 27599-7590 USA; 10 Minority Women Health Alliance (TriStroke), 5409 Olive Road, Raleigh, NC 27606 USA; 11 University of North Carolina Wilmington School of Nursing, 601 S. College Road, Wilmington, NC 28403 USA; 12 Department of Orthopaedic Surgery, Doctor of Physical Therapy Division, & Center for the Study of Aging and Human Development, Duke University, DUMC 104002, Durham, NC 27708 USA; 13 American Heart Association, 3131 RDU Center Drive, Suite 100, Morrisville, NC 27560 USA

**Keywords:** Stroke, Transitions of care, Rehabilitation, Functional status, Pragmatic trial, Patient-centered care

## Abstract

**Background:**

Patients discharged home after stroke face significant challenges managing residual neurological deficits, secondary prevention, and pre-existing chronic conditions. Post-discharge care is often fragmented leading to increased healthcare costs, readmissions, and sub-optimal utilization of rehabilitation and community services. The COMprehensive Post-Acute Stroke Services (COMPASS) Study is an ongoing cluster-randomized pragmatic trial to assess the effectiveness of a comprehensive, evidence-based, post-acute care model on patient-centered outcomes.

**Methods:**

Forty-one hospitals in North Carolina were randomized (as 40 units) to either implement the COMPASS care model or continue their usual care. The recruitment goal is 6000 patients (3000 per arm). Hospital staff ascertain and enroll patients discharged home with a clinical diagnosis of stroke or transient ischemic attack. Patients discharged from intervention hospitals receive 2-day telephone follow-up; a comprehensive clinic visit within 2 weeks that includes a neurological evaluation, assessments of social and functional determinants of health, and an individualized COMPASS Care Plan™ integrated with a community-specific resource database; and additional follow-up calls at 30 and 60 days post-stroke discharge. This model is consistent with the Centers for Medicare and Medicaid Services transitional care management services provided by physicians or advanced practice providers with support from a nurse to conduct patient assessments and coordinate follow-up services. Patients discharged from usual care hospitals represent the control group and receive the standard of care in place at that hospital. Patient-centered outcomes are collected from telephone surveys administered at 90 days. The primary endpoint is patient-reported functional status as measured by the Stroke Impact Scale 16. Secondary outcomes are: caregiver strain, all-cause readmissions, mortality, healthcare utilization, and medication adherence. The study engages patients, caregivers, and other stakeholders (including policymakers, advocacy groups, payers, and local community coalitions) to advise and support the design, implementation, and sustainability of the COMPASS care model.

**Discussion:**

Given the high societal and economic burden of stroke, identifying a care model to improve recovery, independence, and quality of life is critical for stroke survivors and their caregivers. The pragmatic trial design provides a real-world assessment of the COMPASS care model effectiveness and will facilitate rapid implementation into clinical practice if successful.

**Trial registration:**

Clinicaltrials.gov: NCT02588664; October 23, 2015.

**Electronic supplementary material:**

The online version of this article (doi:10.1186/s12883-017-0907-1) contains supplementary material, which is available to authorized users.

## Background

Stroke is a major cause of long-term disability and mortality in the United States, exemplifying a complex chronic disease with high co-morbidity, associated healthcare costs, and readmission rates [1–6]. An estimated 6.6 million Americans aged 20 years and older have had a stroke, and each year approximately 795,000 people experience a new or recurrent stroke [5]. North Carolina (NC) has a particularly high stroke burden and is in the geographic region of the country known as the ‘Stroke Belt’. Stroke mortality in Eastern NC is 40% higher than the national average and hospital admission rates are the highest in the state [7]. African Americans comprise over 20% of the NC population and are more likely to be affected by stroke than their white counterparts, such as having higher post-stroke hospital readmission rates [5, 7, 8].

Stroke survivors experience significant physical, mental and social challenges. Roughly half are discharged directly home from the hospital, where they often realize disabilities not identified during hospitalization and are at risk for complications such as falls, physical deconditioning, aspiration pneumonia, infections, social isolation and depression as well as recurrent stroke [4, 9–16]. Patients with mild post-stroke disability often have undetected physical and cognitive deficits that interfere with function and management of risk factors and medication [17, 18]. Individuals discharged with a clinical diagnosis of transient ischemic attack (TIA) may also have residual deficits and are at increased risk for future stroke [19–21].

Family members who care for stroke survivors often face significant burden [22–24]. Approximately 70% of stroke survivors require assistance with activities of daily living [5]. This assistance is usually provided by family members who are often unprepared and ill-equipped to assume caregiving responsibilities such as providing direct care (e.g., bathing, toileting, mobility assistance, transfers), managing medications, assisting with instrumental activities of daily living, managing stroke survivor emotions and behaviors, communicating with providers, and identifying and accessing community resources. These new responsibilities often lead to overwhelming physical and emotional strain, depressive symptoms, decline in physical and mental health, reduced quality of life, and isolation in the family caregiver [23, 25].

An effective, post-acute care model is needed given the significant impact of stroke on public health, the high risk and complexity of these patients early after discharge, and the strain on caregivers [26, 27]. Currently the U.S. lacks a standard to require hospitals to coordinate community-based follow-up visits with primary or specialty care [26]. The variation in availability, access, referrals, and effectiveness of post-acute stroke medical and rehabilitative care leads to significant gaps in the stroke system of care [28]. These gaps can lead to increased likelihood of readmission, and thus higher healthcare costs. Improved transitions of care from the acute phase to the post-discharge environment may help improve functional status and quality of life for stroke survivors and their caregivers and reduce healthcare costs to society [27]. Stroke morbidity and mortality may also be reduced through effective transitional care, secondary prevention, and rehabilitation early post-stroke [29–33].

Early supported discharge (ESD) is the only evidence-based transitional care intervention found to reduce the negative impact of stroke (i.e., decreased length of stay, accelerated functional recovery, improved patient and caregiver satisfaction) and is the standard of care in the United Kingdom and Canada [24, 31, 34–37]. ESD utilizes a hospital-based, multidisciplinary team of physicians, nurses, therapists, and social workers with stroke expertise to decrease length of stay, anticipate, prevent, and manage stroke complications, improve functional status, and optimize stroke risk factor management, all without increasing caregiver burden [24]. ESD has not been evaluated in the U.S. or non-urban settings and few of its components are in place at 29 stakeholder NC hospitals we surveyed [38].

Our goal was to develop, implement, and evaluate a comprehensive, evidence-based, post-acute care model in real-world practice. Our care model uses elements of transitional care management and ESD evaluated in our prior work with the TRAnsition Coaching for Stroke (TRACS) model that demonstrated reduced readmissions [39]. The intervention was designed to be consistent with the Centers for Medicare and Medicaid Services (CMS) care and reimbursement models for transitional care management [40]. We engaged a range of stakeholders from patients to policy makers, with the goal of making the intervention patient-centered, useful to providers, and lead to greater uptake of results by the larger medical community.

### Study aims

The COMPASS Study was designed to compare the COMPASS care model versus usual post-discharge care on stroke survivors’ self-reported functional status (Stroke Impact Scale-16) 90 days post-stroke hospital discharge. Secondary aims were to determine if the COMPASS care model reduces: i) caregiver strain 90 days post-stroke discharge; ii) all-cause 30- and 90-day readmissions; iii) mortality, health care use (emergency department visits, all-cause re-hospitalization, admission to skilled nursing facilities, admission to inpatient rehabilitation facilities) up to one year after stroke hospitalization; and iv) medication non-adherence.

## Methods

### Objective & study design

The study objective was to evaluate the effectiveness of the COMPASS care model against usual care in diverse hospital settings and patients under real-world clinical conditions. We utilized a cluster-randomized pragmatic trial design in which the unit of randomization was the hospital. This design was chosen as it provides a real-world assessment of our new care model versus usual care. Our design also facilitates rapid dissemination and uptake of successful care strategies and helps health systems prepare for CMS value-based care models [40–43].

### Hospital recruitment

**Fig. 1:**
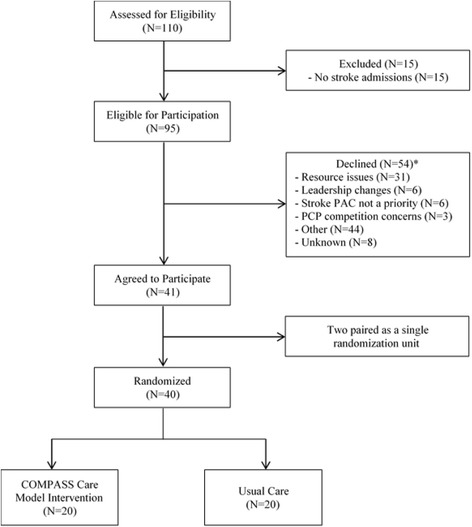
Hospital recruitment and randomization. *Reasons are not mutually exclusive. “Other” reasons include: Decision made at the health system level; bureaucratic issues; decision maker(s) unconvinced of additive value of participation; concerns about sustainability, who should be the on-site principal investigator, and/or IRB/consenting

Hospitals eligible to participate in the COMPASS Study included those that 1) were located in North Carolina; 2) had an emergency department; 3) treated stroke patients; and 4) were able to identify stroke and TIA patients concurrent with care. Recruitment efforts first targeted hospitals in the NC Stroke Care Collaborative (NCSCC) network (*N* = 51) [4]. The NCSCC is a hospital-based, prospective registry of stroke patients and was part of the Centers for Disease Control and Prevention’s Paul Coverdell National Acute Stroke Registry Program through 2014 [4]. It was designed to measure, track, and improve the quality of acute stroke care. The NCSCC hospital network covers 60% of the counties in NC, 80% of the state’s population, and 82% of stroke discharges annually. Recruitment efforts were next extended to all other eligible hospitals in NC. Hospital recruitment lasted for 1 year by which time a total of 110 hospitals in NC had been identified as potentially eligible and invited to participate. Fifteen hospitals did not meet one or more of the inclusion criteria (Fig. 1) because they transferred all stroke patients from the emergency department to another hospital. In addition, 54 hospitals declined to participate in the study. Limited resources (e.g., finances, staff, time) necessary to establish the COMPASS clinic infrastructure and adopt the intervention care model was the primary reason given for non-participation.

**Fig. 2:**
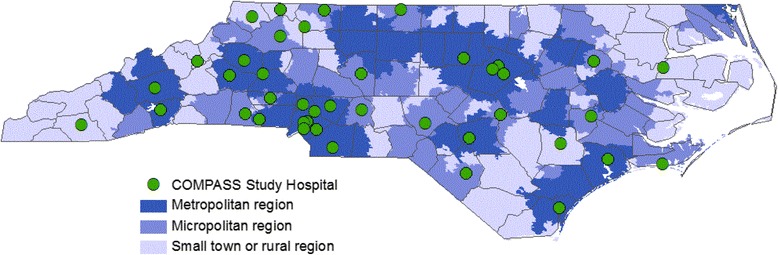
COMPASS Study participating hospitals in North Carolina

Forty-one hospitals (40 randomized units) agreed to participate in the COMPASS study (Fig. 1). Two hospitals were paired as a single randomization unit because their degree of shared staff would have compromised the integrity of independent randomization. Participating hospitals are distributed across urban and rural regions of the state as well as across the three geographic regions of NC, with the majority located in the central Piedmont region (Table 1; Fig. 2) where much of the state’s population resides. Twenty-three sites were Joint Commission certified as a primary or comprehensive stroke center at the time of randomization and 30% had high annual stroke volumes.

**Table 1:** Characteristics of randomized hospital units in the COMPASS Study

	Total (*N* = 40)	Intervention (*N* = 20)	Usual care (*N* = 20)
Joint Commission certified stroke center, n (%)	23 (58%)	12 (60%)	11 (55%)
Critical Access Hospital, n (%)	5 (13%)	3 (15%)	2 (10%)
Geographic region, n (%)			
Piedmont	17 (43%)	9 (45%)	8 (40%)
Western	11 (28%)	6 (30%)	5 (25%)
Eastern	12 (30%)	5 (25%)	7 (35%)
Medical school affiliation, n (%)			
Major	3 (8%)	1 (5%)	2 (10%)
Graduate or limited	5 (12%)	2 (10%)	3 (15%)
None	32 (80%)	17 (85%)	15 (75%)
Annual stroke volume, n (%)			
< 100 discharges	11 (28%)	5 (25%)	5 (25%)
100–299 discharges	17 (43%)	9 (45%)	9 (45%)
300+ discharges	12 (30%)	6 (30%)	6 (30%)
Hospital bed size, n (%)			
< 100 beds	15 (38%)	8 (40%)	7 (35%)
100–300 beds	15 (38%)	6 (30%)	9 (45%)
≥ 300 beds	10 (25%)	6 (30%)	4 (20%)
Urban-rural classification, n (%)			
Rural/Small town	4 (10%)	3 (15%)	1 (5%)
Micropolitan	15 (38%)	8 (40%)	7 (35%)
Metropolitan	21 (53%)	9 (45%)	12 (60%)
30-day Rate for stroke patients, median (IQR)			
Mortality	15.5 (14.2–16.5)	15.6 (13.9–16.6)	15.2 (14.2–16.4)
Readmissions	12.8 (12.2–13.7)	12.8 (12.1–13.9)	12.8 (12.4–13.8)
CMS HCAHPS* 5-Star Quality Rating, median (IQR)			
Overall summary score	3 (3–4)	3 (3–4)	3 (3–4)
Care transition score	3 (3–4)	3 (3–4)	3 (3–4)

*Centers for Medicare and Medicaid Services Hospital Consumer Assessment of Healthcare Providers and Systems

### Randomization

Hospitals were randomized to either receive the COMPASS care model or maintain their usual care. We used a stratified cluster-randomized approach with a block size of two, which maintained balance between treatment groups while also protecting the validity of the randomization process. Only pairs of hospitals were randomized until the end of the study when two hospitals without matches were randomized individually. Hospitals were assigned to one of four strata defined by stroke patient discharge volume and primary stroke center (PSC) certification status (PSC/high-volume; PSC/low-volume; non-PSC/high-volume; non-PSC/low-volume). The study team involved with site selection (RD, SJ) did not have access to the randomization schedule, which was held by a biostatistician (WA) who performed all randomizations and was not involved in site selection. Randomization continued in three waves from April, 2016 through January 2017.

A central IRB was established by Wake Forest University to facilitate oversight of study activities across the 41 participating hospitals. This IRB reviewed and approved the study protocol including the unique aspects of patient consent in this pragmatic trial, discussed later in this paper.

The study period for analysis of the primary and secondary aims (Phase 1) will last until accrual of 6000 participants. At the close of Phase 1, hospitals randomized to usual care will receive the COMPASS care model while hospitals randomized to COMPASS care model in Phase 1 will sustain the model with their own resources (Phase 2). This delayed-start study design allows all enrolled hospitals to eventually receive the intervention [44].

### Participant inclusion & exclusion criteria

The COMPASS Study will enroll 6000 stroke patients discharged home from participating hospitals in Phase 1 (3000 each per randomization arm over ~1 year) and an additional 6000 patients in Phase 2. Patient enrollment began after each hospital was randomized and trained. Eight sites began enrollment in July, 2016, 10 between October–November 2016, and 22 from December 2016–March 2017. Patient eligibility criteria, selected to maximize external validity, are as follows:

1. Aged 18 years of age or older
2. English or Spanish speaker
3. Diagnosed with ischemic stroke, hemorrhagic stroke (excluding subdural or aneurysmal hemorrhage), or TIA; excluding elective carotid endarterectomy procedures
4. Discharged to home (excludes discharges to prison, nursing home, inpatient rehabilitation facility, hospice and comfort measures only)

Hospital staff use a clinical algorithm with standardized definitions for stroke and TIA diagnosis (Additional file 1). Ischemic stroke requires persistence of symptoms for at least 1 h and at least 1 of the following: MRI confirmation of infarct or ischemia, receipt of tPA with or without confirmation on MRI, or high suspicion of cerebrovascular etiology if MRI was negative. Non-traumatic intra-parenchymal hemorrhage requires persistence of symptoms >1 h and CT/MRI evidence of hemorrhage. TIA is defined as a transient episode of neurological dysfunction caused by focal brain, spinal cord, or retinal ischemia without acute infarction. Specifically, TIA diagnosis requires symptoms >5 min in duration with MRI finding negative for acute infarct and absence of suspected stroke mimics (i.e., syncope, complicated migraine, infection, reactivation of old stroke symptoms, delirium, medication reaction, intoxication, angina, and seizure).

### Case ascertainment

Clinical or administrative nursing staff ascertain presumptive stroke and TIA patients concurrently with care by generating a daily list. They query hospital admissions, neurology consults, emergency department logs, and observation units for stroke-related ICD codes and symptoms. Ascertained patients are followed concurrently with care and eligibility is determined using a brief form and clinical algorithm. Patients discharged on a weekend or holiday may be ascertained on the following business day and enrolled retrospectively. The study team performs periodic case ascertainment audits to compare study enrollment against hospital discharge lists.

### Participant enrollment & retention

Designated hospital staff (post-acute coordinator [PAC] at an intervention site or stroke care coordinator at a control site) visit eligible patients prior to discharge. They notify the patient of the hospital’s participation in the COMPASS Study and distribute a study information packet that includes a blood pressure log and site-specific informational brochure describing the hospital’s post-acute care and the COMPASS Study. The brochure also explains the voluntary survey call scheduled for 90 days post-discharge. Hospitals in the COMPASS care model intervention arm also distribute additional information (e.g.*,* individualized risk factor information sheets, refrigerator magnet, business card and contact phone number for the PAC, and an appointment for the COMPASS clinic visit.). Reminder letters are mailed to all participants at 30, 60, and 80 days post-discharge describing the study purpose, available resources for stroke survivor and caregivers, and contact information for study staff. The letter at 30 days includes a COMPASS logo magnet and the 80-day letter includes a copy of the 90-day survey questions to assist with the telephone interview.

### Control group: usual care

Hospitals in the control group continue to implement their current standard of care for stroke patients going home during Phase 1. The study does not bar hospitals from implementing changes over the course of the study. In order to assess transitional care practices prior to the start of the study, and to monitor changes over time, all COMPASS participating hospitals completed a baseline survey. The survey was administered using an electronic data capture tool prior to randomization. Information was captured about the practices and structures in place for care transitions, including availability of telephone and neurology follow-up, and utilization of Transitional Care Management Codes (TCM) billing, post-acute quality metrics, and outcome assessments. A second survey will be completed at the close of Phase 1 in order to assess temporal changes in practice that may have occurred in control sites.

### Intervention group: COMPASS care model

**Fig. 3:**
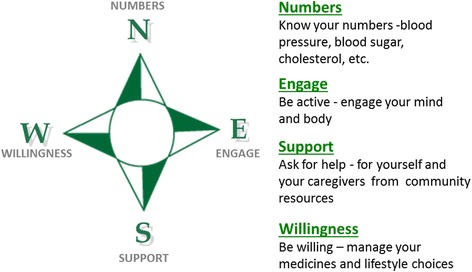
COMPASS key messages - finding the way forward

The COMPASS care model intervention uses a holistic approach and integrates medical and community resources to meet the needs of stroke survivors and caregivers and optimize outcomes. These needs include medical, physical, and social determinants of health, goals of care, and preferences as recommended by the CMS. The COMPASS care model was designed to address these needs by identifying four essential ‘dimensions’ of care to help patients ‘find their way forward after stroke’. These dimensions are depicted as directions on a compass in messaging to patient, caregivers, and practitioners (Fig. 3). The COMPASS care model was developed and refined at the Wake Forest Baptist Transitional Stroke Clinic (the study vanguard site). The model consists of patient follow-up post-discharge and standardized patient and caregiver patient reported assessments. The innovative focal point of the COMPASS care model is real-time generation of an individualized patient electronic care plan (COMPASS-CP™) that also incorporates caregiver support needs and an integrated registry of community resources. The COMPASS-CP was designed to meet CMS requirements for comprehensive care of complex patients and was piloted at the study vanguard site prior to implementation at trial sites [40]. The development of the individualized COMPASS-CP was guided by key patient-centered questions (Table 2) and identifies areas of concern that prohibit maximizing full recovery and provide a solution in the form of education, coaching, and referrals to community-based resources.

**Table 2:** Patient-centered questions that informed design of the COMPASS care plan

Patient-centered question	Incorporation into the COMPASS Care Plan (COMPASS-CP)
What are my health concerns?	The COMPASS-CP template identifies health problems and concerns, both clinical and non-clinical, which could influence health, independence, and recovery
Why is this important to me?	The template explains the importance of each of the “problem” domains based on the results from the 4 assessments. COMPASS’s four dimensions of care are used to relay the importance of targeting the “problem” domains during stroke recovery.
How do I find my way forward?	Recommendations for patient self-management or referrals that are unique to the stroke survivors’ needs are included, consistent with patient goals and preferences

The structure and process of the COMPASS care model are key components of training and implementation in the setting of this pragmatic clinical trial. The structural component consists of a team of practitioners (a registered nurse and an Advanced Practice Provider [APP; who is a nurse practitioner [45], physician assistant [46], or physician]) identified from existing hospital staff, usually those who were already involved in stroke patient clinical care. Their primary functions are to: 1) establish community resource networks representing clinical providers, home health agencies, outpatient therapies, and community agencies; 2) provide input for a local directory of community resources; 3) utilize TCM codes during clinic visits [40]; and 4) provide educational materials focused on helping patients, caregivers and providers understand stroke recovery and prevention.

The intervention processes are:

1. Telephone follow-up within 2 days of hospital discharge
  - Medication reconciliation
  - Assessment of new stroke symptoms and receipt of home health or outpatient services
  - Schedule follow-up appointments with PCP and COMPASS providers
2. Clinic visit within 14 days of discharge during which the patient/proxy undergoes a series of standardized assessments (Table 3)
3. COMPASS-CP development based on the patient’s individualized needs
4. COMPASS-CP implementation:
  - Coordinate and coach patients to incorporate recommended strategies across the 4 COMPASS dimensions of care
  - Make referrals to primary care, specialty care, rehabilitation services, and community services
  - Communicate with primary care, pharmacy, home health, and rehabilitation services
5. Telephone follow-up at 30 and 60 days to evaluate adherence to the COMPASS-CP

**Table 3:** Domains assessed in post-discharge follow-up after stroke

Post-Discharge Follow-Up Call	
• Medication reconciliation	• Home Health/Outpatient Services
• New/Worsening Symptoms	• Caregiver Assistance
• Falls	• PCP follow-up Appointment
• Transportation	• Stroke Clinic Follow-up appointment
Clinic Visit Post-Stroke Functional Assessment	
• Medication Management	• Spasticity
• Financials to Medication Management	• Social Support
• Cognition	• Physical Mobility & Safety
• Depression	• General Health
• Health Literacy	• Upper Extremity
• Access to PCP	• Transportation
• ED/Hospital Readmissions	• General health
• Status of Advance Directive	• Falls
• Stress	• ADL/IADL
Clinic Visit Caregiver Assessment	
• Caregiver Assistance	• Caregiver Stress
• Caregiver Health	
Clinic Visit Advance Practice Provider Assessment	
• Lifestyle Management (alcohol, smoking, drugs,	• Risk factor management (blood pressure, LDL, INR, HgA1c)
• Modified Rankin Scale	• Depression
• Cognition	• Communication
• Physical activity	

**Fig. 4:**
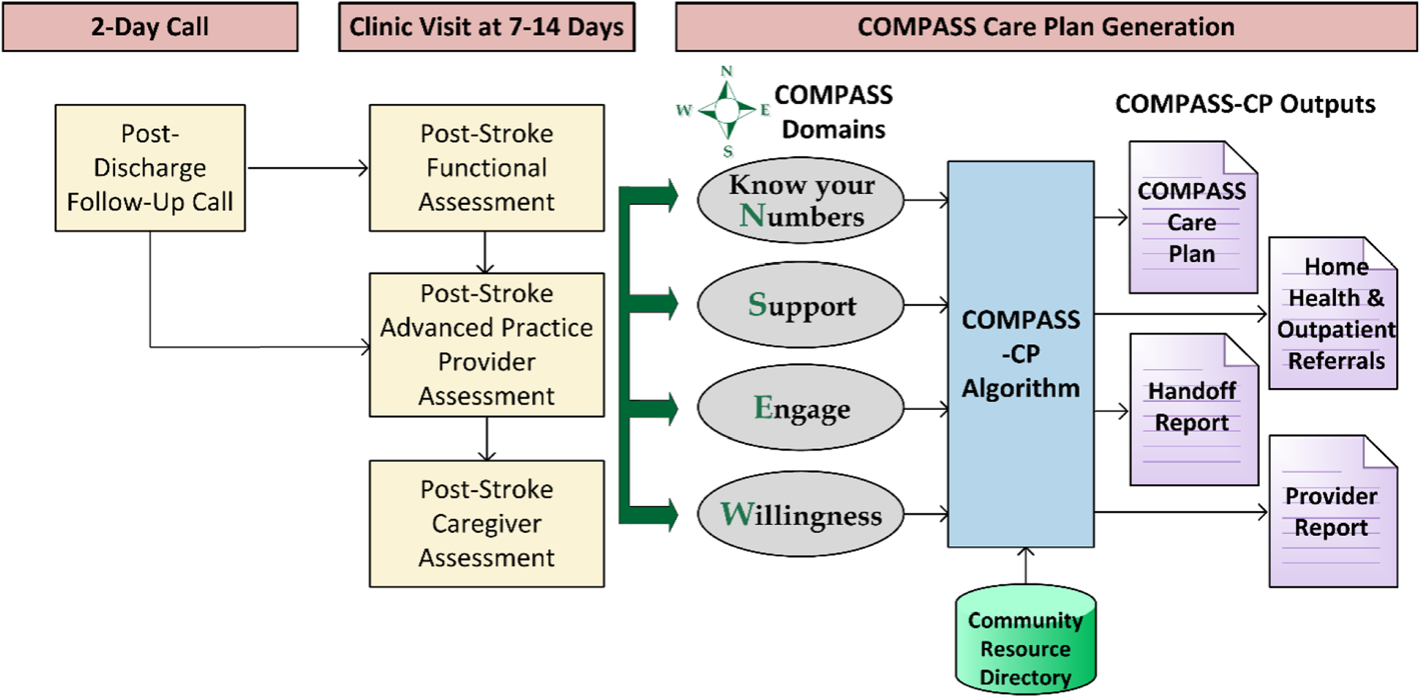
Generation of the patient individualized COMPASS care plan based on inputs from the clinic visit assessments

The PAC and APP conduct 3 standardized assessments at the clinic visits using an iPad application. Patient responses are entered into a web-based data-entry system with *a priori* defined skip logic. Patient-reported outcome measures (PROMs) captured as part of these assessments include, among others, functional and social determinants of health, cognitive status, health data (e.g.*,* blood pressure, medication reconciliation), lifestyle management (e.g.*,* alcohol, smoking, physical activity), and knowledge of stroke risk factors (Table 3) [47, 48]. For patients needing caregiver support, we supplemented the PROMs with self-reported assessment of the caregiver’s role in patient self-management and their ability to provide the necessary support. Algorithms evaluate the data captured during these assessments and identify factors that are likely to influence health, recovery and independence of the stroke survivor across each dimension of care (Fig. 4). These are used to generate the COMPASS-CP, entitled ‘Finding My Way Forward for Health, Independence, and Recovery’ , that is then finalized by the APP (Additional file 2). A printed copy of the COMPASS-CP is provided to each patient and is available electronically to the patient and all providers who manage the patient.

### Implementation monitoring and evaluation

As part of the pragmatic trial we are monitoring intervention fidelity and have developed a set of post-acute quality measures that reflect the core components of the COMPASS intervention. These include receipt of a follow-up phone call within 2 business days, a clinic visit within 14 calendar days, a printed COMPASS-CP during the clinic visit, and home health or outpatient rehabilitation services within 30 days among those who were referred. We hope the results of this pragmatic trial will provide evidence to create national quality standards for post-acute stroke care delivery, which currently do not exist.

In parallel to the pragmatic trial, and with supplemental funding from the Wake Forest Clinical and Translational Science Institute, we are conducting a comprehensive process evaluation of the COMPASS care model implementation guided by the RE-AIM Framework [49]. This analysis will evaluate the Reach, Adoption, Implementation, and Maintenance of the COMPASS intervention while the pragmatic trial will evaluate Effectiveness. Efforts to change clinical practice and deliver evidence-based patient care must be monitored and evaluated to inform broader dissemination. We seek to advance implementation science by identifying individual, organizational, and community factors affecting implementation of the COMPASS care model. For the process evaluation, we will use a mixed methods design. Data will include quantitative data captured from bi-weekly questionnaires to the hospital-based PAC teams that assess perceived barriers to uptake and real-time data on enrollment and performance measures. We will also use qualitative data obtained from interviews with the implementation team and from transcription and coding of bi-weekly phone calls with PAC teams. These data, collected from intervention sites over 1 year, will allow us to identify patient, staffing, and community-level factors that impact intervention uptake, pose challenges to uptake, and influence a health system’s ability to improve performance on pre-defined performance measures.

### Hospital training

All hospital staff were provided training on case ascertainment, enrollment, study design and data quality. Community providers at intervention sites also received comprehensive training of the evidence-based COMPASS care model. Training included site visits for all hospitals and an intensive two-day centralized training “boot camp” for PACs and APPs that equipped them for intervention implementation. Ongoing support is provided with monthly conference calls and distribution of hospital data quality and performance reports that are reviewed with sites monthly to highlight successes and processes that need improvement.

### Outcome assessment

Patient and caregiver outcomes are assessed over a 12-month follow-up period beginning at hospital discharge. The primary outcome is patient functional status 90 days post-stroke discharge that assesses the degree to which stroke-related impairments and disabilities affect the patient’s functional status and quality of life [50]. The SIS-16 is a self-reported questionnaire that can be completed by the patient or a proxy and was selected because it is an outcome that matters to patients, their caregivers, and stroke experts [51, 52]. The Modified Caregiver Strain Index is collected from patient’s caregivers as a secondary outcome [53]. We also assess a variety of secondary patient outcomes using the telephone survey and administrative claims data (Table 4) [53–66]. All surveys are available in English and Spanish.

**Table 4:** Secondary outcomes collected in the COMPASS Study

Patient self-reported outcomes	Outcomes obtained from administrative claims data	Caregiver self-reported outcomes
• General Health Measures	• Hospital readmissions (30- and 90-day)	• Modified Caregiver Strain Index
• Modified Rankin Scale (mRS)	• Emergency department visits	• Caregiver General Health Measure
• Physical Activity	• Hospitalizations: Admissions and hospital days	• Caregiver Support Services
• Patient Health Questionnaire (PHQ-2)	• Skilled nursing facility use	
• Montreal Cognitive Assessment (MoCA) 5-min protocol	• Inpatient rehabilitation	
• 4-Item Morisky Green Levine Scale		
• Secondary Prevention Self-Management		
• Usual care Provider Questions		
• Use of Therapist services		
• Falls and Hospitalizations		
• PROMIS Fatigue Instrument		
• Satisfaction with Care		
• Use of Community Resources		

Trained interviewers assess PROMs through a telephone survey conducted approximately 90 days after hospital discharge. Interviewers are blinded to treatment group and use standardized scripts and interviewing guidelines. We utilize reminder letters, additional phone contacts, mailed surveys, and proxy interviews to increase follow-up rates. Approximately 95 days after patient’s hospital discharge, a self-administered survey is mailed to the individual whom the patient had identified as their primary caregiver. This survey includes the Modified Caregiver Strain Index. If after 3 weeks a completed survey is not received, a reminder telephone call and second mailing are administered. Health care utilization data will be obtained from the CMS Medicare and Medicaid administrative claims and from the North Carolina Blue Cross/Blue Shield Health Insurance and the State Health Care Plan data. These data include claims for hospital visits, emergency department visits, admissions to skilled nursing and inpatient rehabilitation facilities, and hospital use of transitional management billing codes.

### Statistical considerations

Intention-to-treat analyses will be performed at the individual level with adjustment for lack of independence between hospitals. We will use a mixed model to compare the primary endpoint (SIS-16, measured on a continuous scale) between the COMPASS care model and control groups. Although the stratified randomization of hospitals should balance most important hospital-level characteristics between groups, imbalances in patient-level characteristics may exist. Therefore, the proposed mixed model will include both fixed and random effects. The primary analysis model will include two fixed effects, randomization stratum (1 to 4) and intervention (COMPASS care model vs. control), and one random effect (hospital). Sample size and power calculations were based on direct comparisons between the expected changes in outcome means in the two groups, adjusted for the intra-hospital correlation. With a sample size of 6000 recruited from 40 randomization units, we calculate that we will have 83.2% power to detect a 4.17 unit difference in the SIS-16 score for patients in the two groups (assuming a standard deviation of 16.1) [50]. This assumes that 90% of patients will be evaluated at 90 days, an intra-class correlation of 0.036 (based on preliminary data from NCSCC hospitals), and a detectable effect of 0.192 times the within-group standard deviation. The COMPASS Study was also designed to detect differences within subgroups of interest that comprise at least 20% of the overall sample (e.g.*,* stroke subtype, severity, insurance status, geographic area of residence, race, and gender). The intention-to-treat analyses will be complemented with a treatment by protocol analysis, controlling for covariates that differ between those treated per protocol (*i.e*.*,* who received an individualized care plan) and those who were not.

### Informed consent

Because of the barriers to using traditional consent methods in a cluster-randomized pragmatic trial design, [67, 68] we used the broadcasting method. Patients are notified about the study and allowed to ask questions, but there is no option of opting out of participation in the intervention other than seeking care at another hospital [68]. By adopting this method, we minimized patient risk and burden on clinical staff and maximized participation. The COMPASS Study brochure included a way to opt out of the research component (*i.e*.*,* the 90-day survey). Verbal consent was obtained at the beginning of the telephone survey for the collection of those data.

### Stakeholder engagement shaped the study design

Stakeholder engagement shaped our choice for a delayed start design, which provided the best statistical control to assess the intervention’s effects compared with current practice, while also ensuring that all communities would benefit from the trial. Hospital stakeholders (stroke and administrative leadership teams at 29 hospitals) explicitly requested the opportunity to implement the COMPASS care model at some point in the study even if initially randomized to the control group. This design feature supported our hospital recruitment efforts. Patients and caregivers determined the primary outcome, which measures aspects of physical functioning that matter to patients and their families, and is the only widely used measure of stroke outcomes developed from patient and caregiver focus groups [50, 51, 69]. Patients and caregivers also determined the selection of our secondary outcome of caregiver strain. Stakeholders helped develop the informed consent process (*i.e*.*,* timing and language) and study materials. In addition, we convened site-specific meetings with each intervention hospital and their partners to engage local community stakeholders, patients and caregivers and to orient them to the study purpose, intervention and follow-ups, and available learning modules. The community stakeholders included primary care physicians, pharmacists, home health agencies, outpatient rehab providers, Area Agency on Aging representatives, faith leaders, emergency medicine paramedics, and other community partners. Additional information on our methods for engaging a broad base of stakeholders are described elsewhere [70].

## Discussion

This study was designed to evaluate whether implementation of a post-acute stroke care model in real-world clinical practice improves functional status of stroke patients discharged home compared with usual care. Stroke is a high-cost, complex chronic disease that requires extensive post-acute management. Comprehensive post-acute services for stroke require bridging hospital-based acute care with expanded care teams for rehabilitation, primary care management, access to community resources, and caregiver support. Early supported discharge and other transitions of care processes may improve functional outcomes, but uncertainty remains as to the best method to manage stroke patients as they transition to home [26]. The CMS is setting new directions and reimbursements for value-based care for chronic conditions. Stroke may soon be targeted for 90-day bundled payments, which link payments for services rendered during an episode of care encompassing the inpatient stay and post-acute services through 90 days. We have developed a multidisciplinary care model that would meet these new standards.

A pragmatic trial addresses questions of effectiveness, the degree of benefit of a given intervention tested in a real-world setting. There is urgency for such information to evaluate recommended transitional care and comprehensive services for stroke that can be readily implemented into practice. The COMPASS Study will also allow us to systematically evaluate the uptake of the intervention in a diverse environment of hospital systems across North Carolina. At the same time, we employed several strategies from a traditional trial design to measure adherence to the intervention, increase participant retention, and standardize the capture of the primary outcome in order to minimize selection bias. In keeping with a pragmatic design, participants are enrolled with few exclusion criteria and we believe this will contribute to the generalizability of our findings to stroke patients outside of North Carolina. We will be assessing potential modifiers (*e.g*., age, race, sex, insurance status, and stroke severity) and will consider these in drawing inferences to different target populations. Moreover, even though current post-acute care may vary, our pragmatic care model incorporates this variability by tailoring the intervention to each community and individual patient’s needs, connecting patients with local resources. This adaptability further supports generalizability of the COMPASS care model to other clinical settings.

As part of our design, we chose to randomize the hospital and not individual patients within a hospital. This was deemed most appropriate given the system-level nature of the intervention, which made patient-level randomization within a single site not feasible. Group randomization also allowed us to assess feasibility and sustainability of implementing a new care model as the new standard of care for a health system. The cluster-randomized design however, may lead to imbalance in patient characteristics between randomization groups due to the fewer number of randomization assignments.

The primary operational challenge in conducting the study to date was navigating the complexity of rapidly engaging diverse health systems for research. Hospitals and systems were sometimes slow to engage because of the substantial hospital investment of staff and other resources needed to implement an intervention of this scale. The financial support provided by the study is in keeping with its pragmatic nature and is modest compared with compensation offered in many traditional clinical trials. Health systems have many competing priorities and opportunities. The extensive engagement challenges and solutions required to successfully recruit a diverse mix of hospital systems is the topic of a future publication. Initiating all hospitals simultaneously was also a challenge. To address this we randomized and launched sites in waves. This allowed us to direct separate implementation teams in a more focused manner toward subsets of hospitals. This strategy also had the unexpected benefit of allowing us more time to tailor the intervention to each site and to conduct site-specific visits and trainings in addition to the centralized training sessions. Finally, use of a central IRB for streamlining administrative burden was invaluable as was the rigorous organizational structure needed to manage operations.

## Conclusion

This trial addresses a critical gap in post-acute stroke care management. If successful, the COMPASS care model, which has been implemented into current clinical practice in North Carolina, could be scaled to other settings, or targeted for other chronic diseases, and would position health systems for CMS value-based care models [40–43]. This care model could be further expanded to engage primary care and sub-specialty clinics which, in collaboration with hospitals, would facilitate successful patient transitions to health, independence, and recovery.

## Additional files

Additional file 1: Stroke and TIA diagnostic algorithm. (PDF 358 kb)

Additional file 2: Example COMPASS Care Plan. (PDF 583 kb)

## Abbreviations

APP: Advanced Practice Provider
CMS: Centers for Medicare and Medicaid Services
COMPASS: Comprehensive Post-Acute Stroke Services
COMPASS-CP: Comprehensive Post-Acute Stroke Services Care Plan
CT: Computed topography
ICD: International Classification of Diseases
MRI: Magnetic resonance imagine
NC: North Carolina
NCSCC: North Carolina Stroke Care Collaborative
PAC: Post-acute Stroke Coordinator
PROM: Patient-reported outcome measures
SIS: Stroke Impact Scale
TCM: Transitional Care Management
TIA: Transient ischemic attack
tPA: Tissue plasminogen activator
