# Supplementary material for: The Comprehensive Post-Acute Stroke Services (COMPASS) study: design and methods for a cluster-randomized pragmatic trial
Source: BMC Neurol. 2017 Jul 17;17:133. doi: 10.1186/s12883-017-0907-1 (PMC5513078; doi:10.1186/s12883-017-0907-1)
Supplement: Supplementary file 1 — Stroke and TIA diagnostic algorithm. (PDF 358 kb) [file 12883_2017_907_MOESM1_ESM.pdf]

Stroke algorithm for case ascertainment

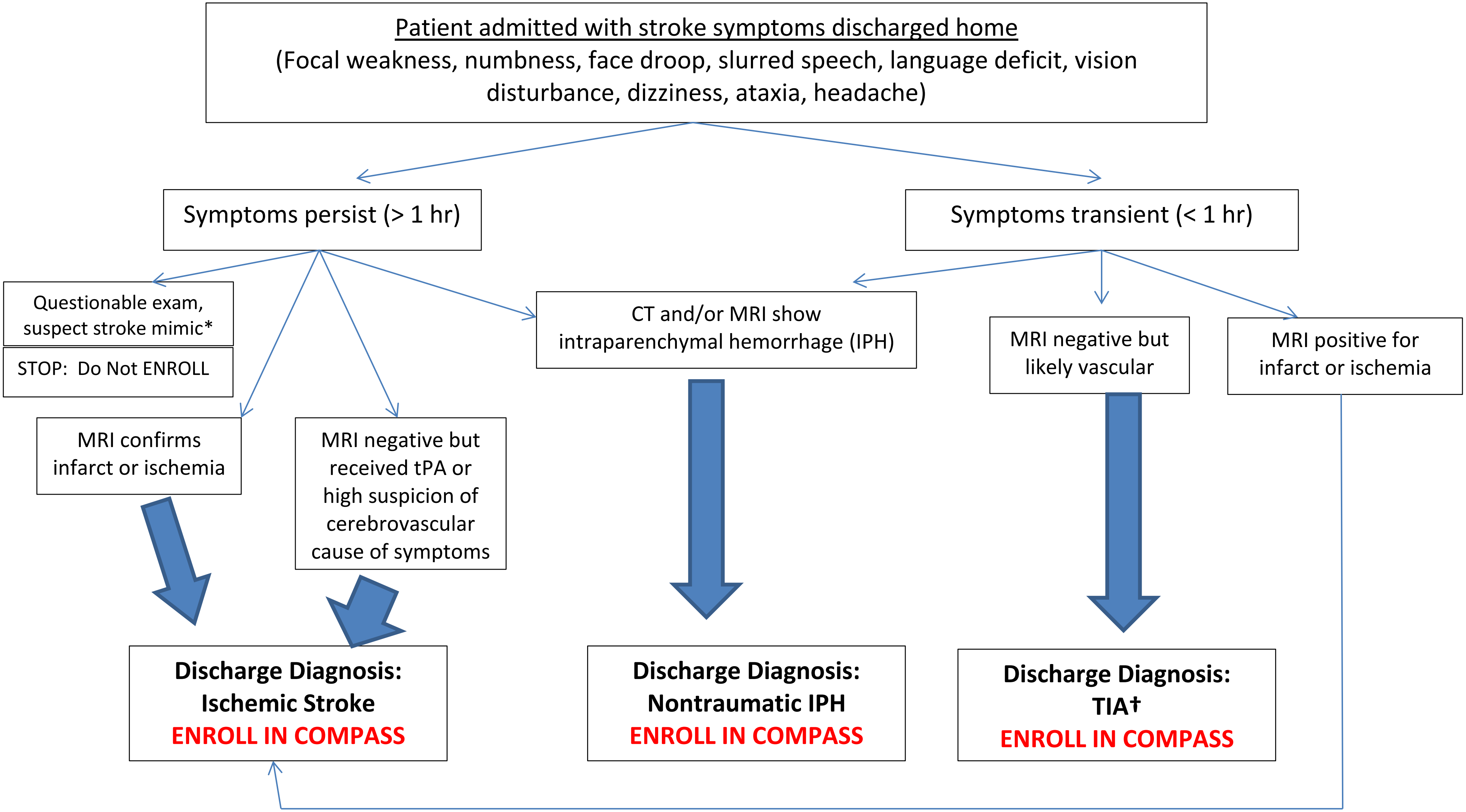

*Discharge diagnosis is TIA vs complicated migraine, syncope, infection, reactivation of old stroke symptoms, medication reaction, delirium

†TIA definition: Transient episode of neurological dysfunction caused by focal brain, spinal cord, or retinal ischemia, without acute infarction.

Inclusion and Exclusion for TIA in COMPASS

TIA

Transient episode of neurological dysfunction caused by focal brain, spinal cord, or retinal ischemia, without acute infarction. [Easton, et al Stroke 2009;40:2276-2293.]

Discharge Diagnosis:
TIA vs another diagnosis

- Complicated migraine
- Infection
- Syncope
- Reactivation of old stroke symptoms
- Delirium
- Medication reaction, side effect, or intoxication
- Angina

STOP: DO NOT ENROLL IN COMPASS

Discharge Diagnosis:
TIA

+

MRI negative for ischemic stroke or infarct

+

Symptoms lasting longer than 5 minutes

*The majority of TIAs last between 5 to 60 minutes, but symptoms could be longer, so there is no upper limit for symptom duration.

ENROLL IN COMPASS
