# Supplementary material for: The Comprehensive Post-Acute Stroke Services (COMPASS) study: design and methods for a cluster-randomized pragmatic trial
Source: BMC Neurol. 2017 Jul 17;17:133. doi: 10.1186/s12883-017-0907-1 (PMC5513078; doi:10.1186/s12883-017-0907-1)
Supplement: Supplementary file 2 — Example COMPASS Care Plan. (PDF 583 kb) [file 12883_2017_907_MOESM2_ESM.pdf]

COMPASS COMPREHENSIVE POST-ACUTE STROKE SERVICES Care Plan

After my stroke, I can GET BETTER! I can recover!

My goal(s) for recovery:
~family

This is my **Care Plan** that is specific to my needs. My COMPASS team will use this plan to help care for me. I can find my way to recovery, independence and health if:

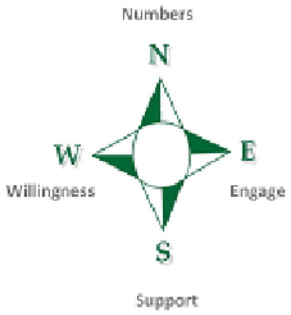

- I Manage My Blood Pressure
- I Am Physically Active – Movement Matters
- I Ask for Help When I Need It
- I Am Willing to Manage My Medications and Lifestyle Choices

My COMPASS team will review this plan with me and will be available for any questions. Please SHARE this Care Plan with all members of your healthcare team which may include primary care provider, nurses, pharmacist, home health and outpatient therapists .

Name of Post-Acute Care Coordinator ____

Telephone Number _________________________

Thank you,
The health care team at ____

Be hopeful and positive!

COMPASS: Finding my Way to Recovery, Independence, and Health

My goal(s):
~family

Name: Gladys Test November 6, 2016 Page 2

COMPASS COMPREHENSIVE POST-ACUTE STROKE SERVICES	What are my concerns?	Why is this important to me?	How do I find my way forward?
**Numbers:** **Know My Numbers. Know My Risks.** NUMBERS N	My Blood Pressure is **180 / 60**	High blood pressure damages the arteries that bring blood to the brain. This can cause another stroke. A blood pressure less than 120/80 is considered normal.	Healthy numbers lead to a healthy life.<br><br>Keeping track of my numbers will decrease my chances of having another stroke.<br><br>I can take my blood pressure every morning and keep a log of my blood pressure numbers.<br><br>I can also check my blood sugar levels daily and keep a log of these numbers as well.
	My hemoglobin A1c level is **8**	Diabetes is a risk factor for stroke. Keeping track of my blood sugar levels can reduce my risk of another stroke. My ideal A1c level is around 7.	
	My LDL (bad) cholesterol level is **100**	A high LDL (bad) cholesterol level puts me at risk for another stroke. My bad cholesterol level should be less than 70.	

COMPASS: Finding my Way to Recovery, Independence, and Health

My goal(s):
~family

COMPASS<br>COMPREHENSIVE POST-ACUTE STROKE SERVICES	What are my concerns?	Why is this important to me?	How do I find my way forward?
**Numbers:**<br>**Know My Numbers. Know My Risks.**<br>NUMBERS<br>N	I do not know all of the risk factors for stroke	There are risk factors I didn't realize could cause another stroke. It's important that I am aware of these risk factors, and my own specific risk factors, so I can make correct lifestyle choices to prevent or manage them.	There are many factors that can put me at a higher risk of having another stroke. The risk factors for stroke are:<br>• High blood pressure<br>• Smoking<br>• Diabetes or high blood sugar<br>• Irregular heartbeat or atrial fibrillation<br>• Heart disease<br>• High cholesterol<br>• Physical Inactivity
**Engage:**<br>**Be engaged to to promote physical activity & safe mobility**<br>E<br>ENGAGE	I have fallen or I am at risk for falling.	I am more likely to fall since I had a stroke. Improving my balance and strength will help decrease my chances of falling and improve my overall independence.	I can decrease my chances of falling by:<br>• Working with a physical therapist in my home or an outpatient clinic.<br>• Attending a falls prevention class<br>• Using appropriate walking aids for support<br>• Having a home safety assessment

COMPASS: Finding my Way to Recovery, Independence, and Health

My goal(s):
~family

COMPASS COMPREHENSIVE POST-ACUTE STROKE SERVICES	What are my concerns?	Why is this important to me?	How do I find my way forward?
**Engage:** **Be engaged with my health care team** E ENGAGE	I need a regular doctor (primary care provider) who knows my medical history and conditions.	A primary care provider will help me monitor my cholesterol, blood pressure, blood sugar and blood thinning.	I can find a primary care provider by:<br>• Using the information given to me in the stroke clinic<br>• Using the information on free clinics if I do not have insurance
	I am not receiving home health or outpatient therapy services, but I may benefit from this.	Skilled therapists can help me improve my strength, balance and ability to safely care for myself and be more independent.	I can ask my primary care provider to make the referrals for home health or outpatient therapy services.

COMPASS: Finding my Way to Recovery, Independence, and Health

My goal(s):
~family

	What are my concerns?	Why is this important to me?	How do I find my way forward?
**Willingness:** **I am Willing to Manage My Medication and Lifestyle Choices**	I am taking a lot of medicines.	Taking multiple medicines after a stroke may be normal. Many of these medicines help to decrease your chance of having another stroke. You should review your medicines with your provider to make sure that all your medicines are still necessary to keep you healthy.	I can share my concerns about my medicines and can get help with how to take them by reviewing them with:<br>• My primary care provider<br>• My Home Health Nurse<br>• My local Pharmacist<br>• My stroke care team<br><br>I may need someone every day to help manage my medicines, fill my pillbox, or remind me so I can take them right.<br><br>I may need a local pharmacy that can provide special services that can help me take my medications correctly.

COMPASS: Finding my Way to Recovery, Independence, and Health

My goal(s):
~family

COMPASS COMPREHENSIVE POST-ACUTE STROKE SERVICES	What are my concerns?	Why is this important to me?	How do I find my way forward?
**Willingness:**<br>**I am Willing to Manage My Medication and Lifestyle Choices**<br>W WILLINGNESS	I do not have anyone to help me manage my medicines, and I may be getting confused about when to take them.	Medicines help to decrease the chance of having another stroke. They are also useful to keeping you healthy. Taking my medicines as directed will give me the best chance for preventing stroke. Skipping doses or stopping my medicines may increase my risk of another stroke.	I can share my concerns about my medicines and can get help with how to take them by reviewing them with:<br>• My primary care provider<br>• My Home Health Nurse<br>• My local Pharmacist<br>• My stroke care team<br><br>I may need someone every day to help manage my medicines, fill my pillbox, or remind me so I can take them right.<br><br>I may need a local pharmacy that can provide special services that can help me take my medications correctly.
	I have not been able to purchase some of my medicines for financial reasons.	Most medicines following stroke need to be filled and started when I get home from the hospital. There may be other medicines for prevention that cost less.	My health care provider or my local pharmacist can help me find cheaper medicines. Other resources include:<br>• Community resources<br>• Financial resources

My recovery and my health require that:

**Numbers**	• **I manage my blood pressure**
**Engage**	• **I am physically active - Movement Matters**
**Support**	• **I ask for help when I need it**
**Willingness**	• **I am willing to manage my medications and lifestyle choices**

For additional information, and to investigate local community resources, visit the COMPASS study website at:

https://www.nccompass-study.org/

You can also visit the American Stroke Association's Life after Stroke web page for more resources to help you move forward in your recovery:

http://www.strokeassociation.org/STROKEORG/LifeAfterStroke/Life-After-Stroke_UCM_308546_SubHomePage.jsp#

You can access this care plan online using the following details:

https://compass.phs.wakehealth.edu/GetMyEcarePlan.cfm
Access code: 3B091D94

Concerns from my CARE plan that could affect my recovery:

I may not know my risk factors that could lead to another stroke.

I have concerns about how I take my medicines.

I have concerns about the cost of my medicines.

I have fallen or I am at risk of falling.

I do not have a primary care provider and I need to get one.

Close monitoring of my medical problems is necessary to prevent readmission to the hospital.

Who should I call for questions or concerns?

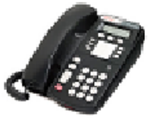

Dial 911 if I experience changes in speech, vision, or walking; or any new sudden onset of one-sided weakness or numbness of the face, arm, or leg.

An easy way to remember the signs and symptoms of a stroke is to think of the word FAST.

Face. Look for an uneven smile.
Arm. Check if one arm is weak or numb.
Speech. Listen for slurred speech.
Time. Call 911 immediately.

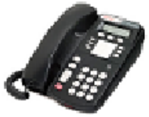

Call my primary care provider for any medical questions that are not related to my stroke.

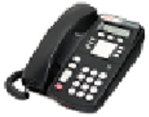

Call the COMPASS team for any questions about my stroke or TIA such as recently prescribed medications, recent hospitalization, home health or outpatient therapy, or community and pharmacy referrals.

COMPASS
COMPREHENSIVE POST-ACUTE STROKE SERVICES

Community Resources: Numbers

**Durham County Department of Public Health<br>Chronic Disease Self Management**	
Organization and Program Information:	(919) 560-7600<br>414 East Main Street<br>Durham, NC 27701<br>http://www.dconc.org

**Independent Living Research Utilization (ILRU)<br>Centers for Independent Living - Alliance of Disability Advocates**	
Organization and Program Information:	(919) 833-1117<br>P.O. Box 12988<br>Raleigh, NC 27605<br>http://www.alliancecil.org/

**Hillsborough Pharmacy & Nutrition<br>Community Care of North Carolina Pharmacy Home**	
Organization and Program Information:	(919) 245-1212<br>110 Boone Square Street #29<br>Hillsborough, NC 27278<br>http://www.hillsboroughpharmacync.com/
